# New Treatment Addressing the Pathogenesis of Psoriasis

**DOI:** 10.3390/ijms21207488

**Published:** 2020-10-11

**Authors:** Michio Tokuyama, Tomotaka Mabuchi

**Affiliations:** Department of Dermatology, Tokai University School of Medicine, 143 Shimokasuya, Isehara, Kanagawa 259-1193, Japan; mabuchi@is.icc.u-tokai.ac.jp

**Keywords:** psoriasis, new treatment, pathogenesis, dendritic cells, Janus kinase inhibitor, sphingosine 1-phosphate receptor 1, Rho-associated kinase 2 inhibitor, aryl hydrocarbon receptor

## Abstract

Psoriasis is an immune cell-mediated inflammatory skin disease. The interleukin (IL)23/IL17 axis plays an important role in the development of psoriasis. The effectiveness of biologic treatments such as tumor necrosis factor (TNF)α inhibitors (infliximab, adalimumab, certolizumab pegol), IL23 inhibitors (ustekinumab, guselkumab, tildrakizumab, risankizumab), and IL17 inhibitors (secukinumab, ixekizumab, brodalumab) have verified these findings. Immune-related cells such as dendritic cells (DCs) and macrophages, in addition to Toll-like receptors and cytokines such as interferon (IFN)α, TNFα, IFNɤ, IL12, IL22, IL23, and IL17, are related to the pathogenesis of psoriasis. Here, we first review new insights regarding the pathogenesis of psoriasis, as it relates to DCs, Langerhans cells, macrophages, the signal transducer and activator of transcription 3 pathway, and aryl hydrocarbon receptor in cutaneous vascular endothelial cells. Based on these findings, we summarize currently available oral treatments and biologics. Furthermore, we describe a new treatment option including Janus kinase inhibitor, tyrosine kinase 2 inhibitor, modulator of sphingosine 1-phosphate receptor 1, and Rho-associated kinase 2 inhibitor.

## 1. Introduction

Psoriasis is a chronic inflammatory cutaneous disease characterized by the formation of scaly, indurated, erythematous plaques. Psoriasis has three principal histologic features: epidermal hyperplasia; dilated, prominent blood vessels in the dermis; and an inflammatory infiltrate of leucocytes, predominantly into the dermis [1]. Not only limited to the skin, psoriasis also affects joints and nails. Psoriasis is mainly divided into three clinical types: psoriasis vulgaris, psoriatic arthritis, and generalized pustular psoriasis (GPP). Psoriasis often coexists with other systemic disorders including obesity, hypertension, hyperlipidemia, diabetes, metabolic syndrome, cardiovascular disease, which is called psoriatic march or inflammatory march, and chronic kidney disease [2,3]. Psychiatric disorders, psychosocial distress such as depression are also seen in psoriatic patients [4].

**Figure 1:**
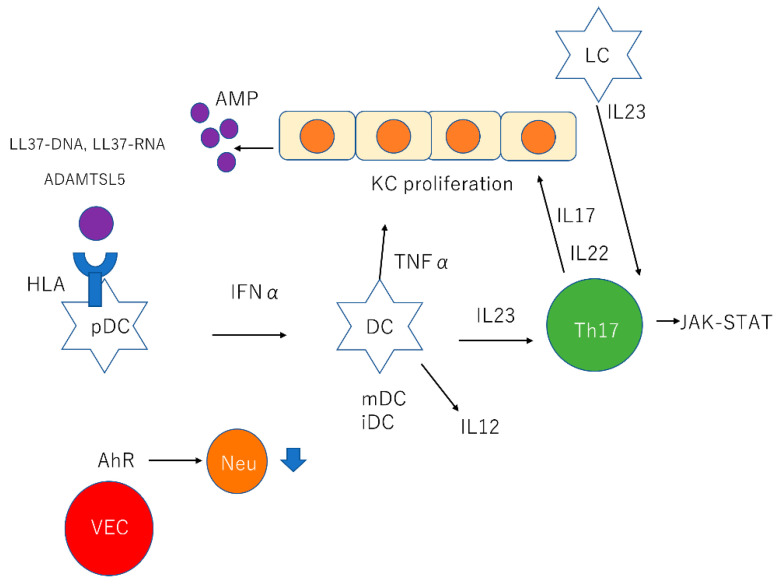
Summary of pathogenesis of psoriasis. KCs produce AMPs such as β-defensins, S100 proteins, and cathelicidin. LL37 and ADAMTSL5 are considered to be as autoantigens. LL37–DNA complexes stimulate pDCs, and pDCs secrete IFNα. LL37-RNA complexes stimulate mDCs and mDCs produce TNFα, IL23, and IL12. IL23 stimulates Th17 cells and Th17 cells produce IL17 and IL22 through the JAK-STAT pathway. LCs also secrete IL23 and stimulate Th17 cell. AhR in cutaneous VECs downregulate neutrophil recruitment. KC: keratinocyte; AMP: antimicrobial peptides; ADAMTSL5: A disintegrin and metalloprotease domain containing thrombospondin type 1 motif-like; pDC: plasmacytoid dendritic cell; IFN: interferon; mDC: myeloid dendritic cell; iDC: inflammatory dendritic cell; TNF: tumor necrosis factor; IL: interleukin; JAK: Janus kinase; STAT: signal transducer and activator of transcription; LC, Langerhans cell; AhR: aryl hydrocarbon receptor; VEC: vascular endothelial cell; Neu: neutrophil; HLA: human leukocyte antigen.

**Figure 2:**
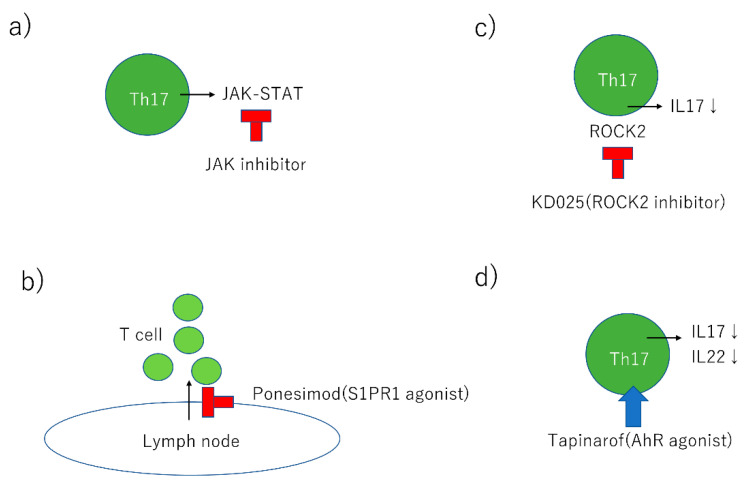
Summary of new treatment. (**a**) JAK inhibitor blocks the JAK-STAT signal pathway in Th17 cells. (**b**) Ponesimod, a selective S1P1 agonist, induces sequestration of lymphocytes into lymph nodes and decreases peripheral lymphocyte counts and trafficking of lymphocytes to peripheral tissues. (**c**) KD025, ROCK2 inhibitor reduces IL17 secretion in Th17 cells. (**d**) Tapinarof, AhR agonist reduces IL17 and IL22 in Th17 cells. JAK: Janus kinase; STAT: signal transducer and activator of transcription; S1PR1: sphingosine-1-phosphate receptor 1; ROCK2: Rho-associated kinase 2; IL: interleukin; AhR: aryl hydrocarbon receptor.

These comorbidities and symptoms affect the choice of treatments. The pathogenesis of psoriasis involves antimicrobial peptides (AMPs), dendritic cells (DCs), tumor necrosis factor (TNF)α, interleukin (IL)23, Th17, IL17, IL22, and signal transducer and activator of transcription (STAT)3 (Figure 1, Table 1). In this review, we focus on the development of psoriasis and summarize new treatments based on new insights (Figure 2, Table 2).

## 2. Pathogenesis of Psoriasis

### 2.1. AMPs

AMPs are composed of 12–50 amino acids, have positive charge, amphipathic structure, and play important roles in host protection by killing pathogenic microorganisms including bacteria, protozoa, fungi, and viruses [5,6]. AMPs also affect host inflammatory responses by acting as chemotactic agents, angiogenic factors, and regulators of cell proliferation in vertebrates [6]. In psoriasis, certain AMPs, including β-defensins, S100 proteins, and cathelicidin, are highly expressed and secreted by keratinocytes, neutrophils, and macrophages in response to injury and cytokine stimulation (Figure 1) [7].

The defensins are cationic microbicidal peptides separated into three categories; defensins α, β, and θ [8]. The α-defensins are further separated into six subtypes designated as human neutrophil peptides (HNPs) 1–6, and HNPs 1–3 are present in the scales of psoriatic lesions [9]. The β-defensins are separated into four subtypes designated as human β-defensins (hBDs) 1–4. TNFα and IFNɤ induce hBDs 2 and 3, which are highly present in the psoriatic scales, and IL17A and IL22 also induce hBDs 2 in keratinocytes [6]. The role of defensins in the pathogenesis of psoriasis is not yet fully understood [10].

The S100 proteins comprise a family of low-molecular-weight (9–13 kDa) proteins [11]. S100A7 (psoriasin), S100A8 (calgranulin A), S100A9 (calgranulin B), S100A12 (calgranulin C), and S100A15 are highly expressed in psoriasis [8]. The combination of IL22, IL17A, IL17F, and keratinocytes synergistically induced the expression of β-defensin 2, S100A9, S100A7, and S100A8 [12]. S100A7 (psoriasin) is reported as a potent and selective chemotactic inflammatory protein for T cells and neutrophils in psoriasis [13].

Cathelicidin LL37 is the C-terminal peptide fragment derived from hCAP18 [14]. Plasmacytoid DCs (pDCs) recognize self-DNA through TLR9 and LL37 is the key factor that mediates pDCs’ activation in psoriasis [15]. Furthermore, keratinocytes exposed to LL37 and self-DNA produce type I IFN, which is related to the development of psoriasis (Figure 1) [16].

LL37 bound to RNA stimulates pDCs through TLR7 and LL37-RNA complexes act on myeloid DCs (mDCs) through TLR8 [7,10,16]. Activated mDCs migrate into draining lymph nodes and produce TNFα, IL23, and IL12. Slan+ monocytes also respond to LL37–RNA activation and produce TNFα, IL23, and IL12 (Figure 1) [17]. IL23 and IL12 induce naïve T cell to Th17 and Th1 cell subsets, respectively [7].

An in silico docking study by Mabuchi et al. confirmed that multiple 9-mer peptides derived from LL37 exhibit high binding affinities for HLA-C*06:02 molecules, and they proposed a mechanism for the interaction between LL37 HLA-C*06:02 complexes and T cells via T-cell receptors [18].

### 2.2. A Disintegrin and Metalloprotease Domain Containing Thrombospondin Type 1 Motif-Like 5 (ADAMTSL5)

The melanocyte-derived protein ADAMTSL5 has been identified as an autoantigen [19]. Intra-epidermal CD8 T cells recognize ADAMTSL5 on melanocytes in association with HLA-C*06:02 [19]. Keratinocytes produce ADAMTSL5 with IL17 stimulation and CXCL1, which is a neutrophil chemoattractant and melanocyte growth factor, induce ADAMTSL5 expression [19]. In psoriasis, the number of melanocytes is increased and T cells including cytotoxic T cells co-localize with these melanocytes [20]. However, it is suggested that melanocytes are likely targets of the non-cytotoxic CD8+ T cell-mediated autoimmune response because the number of melanocytes increase, but melanocytes do not show signs of cell death in psoriasis [21]. The ADAMTSL5 expression pattern mirrors the pattern of T cell infiltration and DC aggregation in the superficial dermis in psoriasis, which is similar to LL37 [22]. The expression of ADAMTSL5 and LL37 with DCs, neutrophils, macrophages, and T cells in psoriasis significantly decreases after treatment of IL17 or TNFα blocker [23,24]. This suggests that ADAMTSL5 and LL37 are presented to autoreactive CD4+ T cells by HLA-class II molecules and to CD8+ T cells by HLA-Cw6*02, which are expressed on the surface of antigen-presenting cells within the dermal lymphoid tissue structures [19,24]. A synthetic ADAMTSL5 peptide increases the frequency of CD8 T cells expressing IL17A and IFNɤ among the peripheral blood mononuclear cells in psoriasis patients, but the same effect is not found in healthy individuals [19].

### 2.3. DCs

The skin contains a complex network of DCs mainly composed of epidermal Langerhans cells (LCs), bone-marrow-derived dermal conventional DCs (cDCs), pDCs, and inflammatory DCs (iDCs) [25]. As the prominent cellular source of IFNα, TNFα, IL12, and IL23, DCs play an important role in psoriasis [25].

pDCs originate in the bone marrow and migrate to the skin under pathologic conditions [25]. pDCs recognize viral nucleic acids and produce large amounts of type I IFNs in response to endosomal Toll-like receptors (TLRs), such as TLR7 and 9 [26,27,28,29]. Inappropriate recognition of self-nucleic acids by pDCs results in producing IFNα and initiating psoriatic inflammation [29,30]. The AMPs such as LL37, human β-defensin (hBD) 2, hBD3, and lysozyme are able to condense self-DNA or RNA into particles that are endocytosed by pDCs, leading to activation of TLR7, 8, and 9 in psoriasis [29,30,31]. In psoriatic inflammation, IFNα is considered to act as an upstream cytokine along the IL23/IL17 axis [32]. IFNα strongly activates immature cDCs to secrete IL12, IL15, IL18, and IL23 [33]. In addition, IFNα induces the rapid differentiation of human monocytes into iDCs and polarizes CD4+ T cells into Th1 and Th17 cells [34,35]. Furthermore, IFNα also enhances the reaction to IL22 on epidermal keratinocytes by upregulating IL22 receptor expression [36].

Under healthy conditions, cDCs are also associated with maintaining immune tolerance through depletion of autoimmune T cells, expressing anti-inflammatory cytokines and inhibitory receptors including IL10, transforming growth factor (TGF)β, and IL27 and prompting the homeostasis of regulatory T cells (Tregs) [37,38]. Dysregulation of this tolerance mechanism has been found in many autoimmune diseases such as psoriasis [39,40]. By inducing tolerogenic DCs, α-melanocyte-stimulating hormone ameliorates psoriasis by promoting Treg expansion and downregulating the proliferation of human and murine Th17 cells and cytokine production [41]. cDCs are activated by multiple cytokines such as IL6, TNFα, IFNα, and also LL37–RNA complexes [25]. Activated cDCs produce a mass of inflammatory cytokines such as IL12 and IL23, which are key mediators in psoriasis [25].

iDCs are considered to be derived from monocytes and have the ability to polarize T cells to Th1, Th2, and Th17 and produce cytokines such as IL1, IL6, TNFα, IL12, IL22, and IL23 [27]. In psoriasis, TNFα, and inducible nitric oxide synthase (iNOS)-producing DCs (Tip-DCs) and 6-sulfo LacNAc DCs (slanDCs) are reported as iDCs, and they induce T cells to secrete IL17, IL22, TNFα, and IFNɤ [14]. SlanDCs are also stimulated by TNFα and produce IL12, IL23, IL1β, and IL6 [17].

The number of LCs in psoriasis is likely to be variable, as it can increase [42], decrease [43], or remain the same [44]. Subsequent experiments demonstrated that increased expression of IL17 is responsible for impaired LC migration in the uninvolved skin of patients with psoriasis [45]. Recent studies demonstrated that activation of the STAT3 pathway in keratinocytes and TLRs in LCs can induce LCs to produce IL23 and monocyte-derived LCs (mLCs) exhibit a more powerful ability to secrete IL23 when compared with resident LCs (rLCs) (Figure 1) [44,46]. Furthermore, LCs are in close contact with T cells in patients who achieved almost complete remission after anti-TNFα treatment, and they retain a higher capacity for secreting IL23 when compared with healthy volunteers following TLR7/8 stimulation in vitro [25]. This emphasizes that LCs may be associated with recurrence of psoriasis, although this ability is to a small extent comparable with progressive stage psoriasis [44]. Recent studies have shown that LCs indeed produce IL23, and the close proximity to pathogenic T cells suggests that LCs may as be associated with the pathogenesis of psoriasis as other DCs [25]. However, a negative regulatory role of LCs has been observed. The function of LCs in psoriasis varies, and LCs may function as the gate that regulates the degree of inflammatory responses in the disease [25].

### 2.4. IL23/IL17 Axis

IL17 is produced by Th17, Tc17, innate lymphoid cells, and ɤδT cells in the skin [47,48]. IL23 expressed by dermal DCs drives IL17 production [49]. IL17 is produced, as well as TNF, IL26, and IL29 (IFNλ1), under specific situations such as psoriasis autoantigens and/or certain environmental stimuli (e.g., trauma or infection) [50]. These cytokine signals create a feed forward inflammatory response in keratinocytes by activating CCAAT enhancer-binding protein (C/EBP) β or δ, STAT1, and nuclear factor kB [49]. This feed-forward response accelerates the development of psoriasis [49]. IL17 collaborates with TNF to potentiate IL17-induced transcription of several proinflammatory genes (e.g., TNF, IL1β, IL6, and IL8) [51]. They activate mDCs and promote the differentiation of Th17 cells in the skin and draining lymph nodes [52].

IL17 regulates epidermal hyperplasia indirectly by activating STAT3 and promotes keratinocytes to induce IL19, IL36 [50]. IL22, which is mainly produced by Th17 and possibly IL20, also activates STAT3 and induces epidermal hyperplasia (Figure 1) [53]. In the upper spinous and granular layers of the epidermis of psoriatic lesions, IL17-induced transcription factors (e.g., C/EBPβ or C/EBPδ) and keratinocyte-derived gene products, including S100A7/8/9, hBD2, lipocalin-2, and CCL20 are increased [49,54]. IL17 in psoriasis has the ability to recruit neutrophils and macrophages by inducing keratinocytes to produce CXCL1, CXCL2, CXCL3, CXCL5, and CXCL8 (i.e., IL8) [46]. IL17A, IL22, and TNF also stimulate CCL20 expression in keratinocytes [55]. CCL20 attracts CCR6+ cells such as mDCs and Th17 cells and sustains the inflammatory response through a positive chemotactic feedback loop [55]. Keratinocytes in psoriasis produce platelet-derived growth factor, angiopoietin-2, and vascular endothelial growth factor, and these growth factors result in erythematous skin lesions [56].

### 2.5. Aryl Hydrocarbon Receptor (AhR)

The AhR is a cytosolic ligand-activated receptor and transcription factor, which is widely expressed in the skin cells [57,58]. Endogenous and exogenous molecules and dioxins are known as ligands of AhR [57,58]. AhR activation induces oxidative stress through CYP1A1 and neutralizes oxidative stress through the nuclear factor-erythroid 2-related factor-2 (NRF2) transcription factor [59]. Furthermore, AhR regulates the balance of the Th17/22 system, which is important for developing psoriasis [59]. AhR agonists decreased IL-23 receptor, Th17 master transcription factor retinoic acid-related orphan receptor C (RORC) and the number of Th17 cells [60].

In addition, AhR in cutaneous vascular endothelial cells (VECs) also plays an important role in the development of psoriasis [61]. Zhu et al. found that AhR in cutaneous VECs downregulates neutrophil recruitment through adhesion molecule ICAM-1 in psoriasis using specific AhR knockout mice (Figure 1) [61].

## 3. Treatment

### 3.1. Currently Available Oral Systemic Therapy

#### 3.1.1. Retinoids

Retinoids are derivatives of vitamin A and bind to nuclear receptors, retinoic acid receptors, and retinoid X receptors, which regulate gene transcription such as IL6 [6]. Retinoids induce keratinocyte differentiation and reduce epidermal hyperplasia, leading to a slowing of cell reproduction [62]. Etretinate, which is a second-generation retinoid, is used in Japan, and acitretin, which is an active metabolite of etretinate, has a shorter half-life, and is eliminated more rapidly than etretinate, is used in many countries [62]. The Psoriasis Area Severity Index 75 (PASI75) response to acitretin 25, 35, and 50 mg/day groups at week 12 were 47%, 69%, and 53%, respectively [63]. The main adverse effects were dry mouth, cheilitis, pruritus, teratogenicity, and elevations in serum lipid and liver enzymes [62,63].

#### 3.1.2. Methotrexate

Methotrexate (MTX) inhibits dihydrofolate reductase (DHFR), an enzyme that participates in the tetrahydrofolate synthesis. MTX induces inhibition of purine, methionine, and thymidylate synthesis and inhibits DNA synthesis [64]. Low-dose MTX may have anti-inflammatory effects, including increased adenosine levels, and modulates immune cells [64]. The PASI75 response to MTX at week 12 was 45.2% [65]. The main adverse events were nausea, vomiting, mouth ulcers, upper respiratory infection, abnormal liver function tests, and interstitial lung disease [65].

#### 3.1.3. Cyclosporine A

Cyclosporine A (CyA) is a calcineurin inhibitor. CyA forms a complex with cyclophilin and blocks phosphatase activity of calcineurin and decreases the production of inflammatory cytokines including in T cells [6,66]. The PASI75 response to CyA 5 mg/kg and 2.5 mg/kg at week 10 to 16 was 50–97% and 28–85%, respectively [66]. The main adverse effects are nephrotoxicity, hepatotoxicity, hypertension, an increased risk of infection, and lymphoma [66].

#### 3.1.4. Apremilast

Apremilast is a selective inhibitor of the enzyme phosphodiesterase 4 (PDE4), which breaks down cyclic adenosine monophosphate (cAMP). PDE4 is the main enzyme in immune cells such as T cells, macrophages, and epithelial cells [6]. Through PDE4 inhibitors, the level of cAMP increases and cAMP downregulates proinflammatory cytokine including TNFα and upregulates anti-inflammatory cytokine including IL10. The PASI75 response to apremilast at week 12 was 33.1% [67]. The main adverse events are diarrhea, vomiting, and depression [67].

### 3.2. Currently Available Biologic Therapy

#### 3.2.1. TNFα Inhibitors

Infliximab, adalimumab, and certolizumab pegol are currently available for the treatment of psoriasis as TNFα inhibitors. Certolizumab pegol is an Fc-free, PEGylated TNFα inhibitor. It does not bind the neonatal Fc receptor for IgG (FcRn) and consequently shows minimal placental transfer from mothers to infants [68]. At week 10, PASI 75 response rates for infliximab at 5 mg/kg dose were 80% [69]. At week 16, PASI 75 response rates for adalimumab at 40 mg after an initial 80 mg dose were 80% [70]. At week 16, PASI 75 response rates for certolizumab pegol at 400 mg were 80.1% [71]. The main severe adverse events were reactivation of hepatitis B and C, tuberculosis, drug-induced lupus, demyelinating central nervous system disorders, and paradoxical reactions such as psoriasis and psoriasiform skin lesions [69,70].

#### 3.2.2. IL23 Inhibitors

Ustekinumab, guselkumab, risankizumab, and tildrakizumab are currently available for the treatment of psoriasis as IL23 inhibitors. At week 12, PASI 75 response rates for ustekinumab at 45 mg and 90 mg were 67.5% and 73.8%, respectively [72]. At week 16, PASI 75/90/100 response rates for guselkumab at 100 mg were 91.2%/73.3%/37.4% and for risankizumab at 150 mg were 90.8%/74.8%/50.7% [73,74]. At week 28, PASI 75/90/100 response rates for tildrakizumab at 100 mg were 77%/54%/23% [75]. The main adverse effects were nasopharyngitis, upper respiratory tract infection, headache, and tiredness [72,73,74,75].

#### 3.2.3. IL17 Inhibitors

Secukinumab, ixekizumab, and brodalumab are currently available for the treatment of psoriasis as IL17 inhibitors. At week 12, PASI75/90/100 response rates for secukinumab at 300 mg were 77.1%/54%/24%, for ixekizumab at 80 mg after an initial 160 mg were 90%/70%/40%, and for brodalumab at 210 mg were 83%/70%/42%, respectively [76,77,78]. The main adverse events were candidiasis, neutropenia, inflammatory bowel disease, and depression and risk of suicide in brodalumab [76,77,78].

### 3.3. RORγt Inhibitors

Retinoic acid receptor-related orphan nuclear receptor gamma t (RORγt or RORc2) is a key transcription factor for Th17 cell differentiation [4]. Inhibiting RORγt activity is thought to be a promising strategy for the treatment of psoriasis [4]. A phase 2a clinical trial of RORγt inhibitor VTP-43742 in psoriatic patients was conducted [79]. A significant reduction in the psoriasis area severity index (PASI) score relative to the placebo was observed in the 350 mg (24%) and 700 mg (30%) dose groups [79]. However, reversible transaminase elevations were observed in the 700 mg dose group in four patients. Because of this liver toxicity, the initially planned third clinical trial of VTP-43742 was discontinued [79]. Although the phase 1 clinical trial of GSK2981278 ointment (selective RORγ inverse agonist) showed a lack of efficacy, new topical RORγt inhibitors may be a potential candidate for the treatment of psoriasis [80,81].

### 3.4. IL36 Receptor Antagonist

IL36 is an IL1 superfamily member and plays an important role for recruiting and activating neutrophils and Th17 cells in psoriasis [82]. Loss-of-function mutations have been found in *IL36RN*, which encodes an IL36-receptor antagonist, in some GPP patients [83]. In the phase 1 clinical trial, an intravenous single dose of BI 655130, a monoclonal antibody against the interleukin-36 receptor showed good efficacy regardless of the presence of the *IL36RN* mutation over a 20-week period [83].

### 3.5. Janus Kinase (JAK) Inhibitors

Type 1 and 2 cytokine receptors strongly depend on the JAK and STAT pathways [84]. The JAK family is intracellular protein tyrosine kinase and includes JAK1,2,3, and TYK2, and the STAT family includes STAT1, 2, 3, 4, 5a, 5b, and 6 [84]. The functions of each JAK and STAT are different [84]. The IL23 receptor, which is important in psoriasis, is associated with JAK2, TYK2, and STAT3 [84]. JAK inhibitors are currently being tested in clinical trials for the treatment of psoriasis (Table 2) [85].

Tofacitinib, which is a first-generation JAK inhibitor, primarily targets JAK3, JAK2, and JAK1 [85]. In the phase 3 studies, the PASI75 response to tofacitinib at weeks 16–24 was 39.5–54.3% (5 mg twice daily) and 59.2–81.1% (10 mg twice daily), as compared to 5.6–12.5% for the placebo [86,87,88,89]. During the study, increased circulating total cholesterol, low-density lipoprotein cholesterol, high-density lipoprotein cholesterol, and creatinine phosphokinase levels and decreased blood hemoglobin and lymphocyte counts were observed [85]. Serious adverse events including angina pectoris, pyelonephritis, urosepsis, and atrial fibrillation were observed in three patients [90]. Nasopharyngitis, sinusitis, upper respiratory tract infection, back pain, and headache were observed as adverse events [90]. Now, new JAK inhibitors such as JAK1/JAK2 inhibitor baricitinib are under clinical trial and exhibited good results [91].

The TYK2 inhibitor BMS-986165, which is a more selective JAK inhibitor than first-generation candidates, showed efficacies of 75% (12 mg daily), when compared to 7% in the placebo group in terms of PASI75 response at week 12 of treatment in a phase 2 clinical trial [85].

The TYK2/JAK1 inhibitor PF-06700841 directly suppresses TYK2-dependent IL12 and IL23 signaling, and JAK1-dependent signaling in cells such as T cells and keratinocytes [92]. PF-06700841 improves clinical symptoms of chronic plaque psoriasis by inhibiting proinflammatory cytokines that require TYK2 and JAK1 for signal transduction [92]. Topical PF-06700841 is now under investigation in a phase 2 trial [93].

### 3.6. Sphingosine-1-Phosphate (S1P) Agonist

Sphingosine is created from ceramide, which is composed of membrane lipids, through ceramidase [94]. S1P is created from sphingosine through sphingosine kinase [94]. S1P is a lipid mediator and associated with cellular proliferation, survival, migration, inflammation, immune cell trafficking, angiogenesis, vascular integrity, and adhesion in the immune and vascular systems [95]. S1P acts on five specific G protein-coupled receptors named S1P receptor (S1PR)1–5 [95].

S1PR1 is expressed on lymphocytes and controls their egress from thymus and secondary lymphoid organs [96,97]. S1PR1 modulators induce internalization of this receptor and the majority of circulating lymphocytes are sequestered in lymph nodes, decreasing peripheral lymphocyte counts and trafficking of lymphocytes to peripheral tissues (Figure 2) [98,99]. Furthermore, it has been reported that S1P inhibits the growth of epidermal cells, induces differentiation of keratinocytes, and shows antiproliferative and anti-inflammatory effects in mouse models of psoriasis [100,101,102,103,104].

Ponesimod (ACT-128800) is an orally selective S1PR1 agonist, which blocks the egress of T cells from lymphoid organs [105,106]. Ponesimod is excreted within 1 week after discontinuation, and this rapid elimination is beneficial in cases including vaccinations and pregnancy [107,108].

In the phase 2 clinical trial, the PASI75 response to ponesimod by week 16 was 46.0% (20 mg) and 48.1% (40 mg), as compared to 13.4% for the placebo. By week 28, PASI75 was achieved in 71.4% (20 mg) and 77.4% (40 mg), respectively [108]. During the trial, dyspnea, increased liver enzyme concentrations, headache, nasopharyngitis, bradycardia, pruritus, and dizziness were observed [108]. However, the phase 3 clinical trial of ponesimod in psoriasis has not been started yet, possibly because severe side effects such as lymphopenia, bradycardia, and dyspnea were observed in other S1PR1 modulators [108,109].

### 3.7. Rho-Associated Kinase (ROCK2) Inhibitor

Rho family kinases, consisting of ROCK1 and ROCK2, are serine–threonine kinases activated by Rho GTPases and mediate the phosphorylation of downstream targets in cells [110,111]. KD025 is adenosine triphosphate (ATP) competitive and 100-fold more selective for the ROCK2 isoform when compared with ROCK1, with no significant activity against 300 other intracellular kinases and surface receptors [111,112]. Recent studies showed that oral administration of a selective ROCK2 inhibitor (KD025) in healthy subjects decreases IL17 and IL21 secretion induced by ex vivo stimulation (Figure 2) [111]. Moreover, targeted ROCK2 inhibition shifted the balance between proinflammatory and immunosuppressive T-cell subsets through concurrent regulation of STAT3/STAT5 phosphorylation [113,114].

In the phase 2 clinical trial, KD025 significantly reduced both IL17 and IL23 levels. KD025 also decreased epidermal thickness, K16 expression, and T-cell infiltration in the skin [114]. In addition, the PASI50 response to KD025 (200 mg twice daily) at week 12 was 71% [114]. In contrast, KD025 significantly increased levels of the immunosuppressive cytokine IL10, but TNFα and IL6 levels were not changed [114]. ROCK2 expression is induced during Th17-skewing conditions and regulates IL17 secretion through a STAT3/IRF4/RORγt-dependent mechanism in mice and humans [111,115].

### 3.8. The AhR Agonist, Tapinarof

Tapinarof (GSK2894512; 5-[(E)-2-phenylethenyl]-2-(propan-2-yl)benzene-1,3-diol, previously WBI-1001) is a naturally derived polyphenol produced by bacterial symbionts of entomopathogenic nematodes and an AhR agonist [116,117]. In ex vivo human skin, tapinarof reduced Th17 cytokines such as IL17A, IL17F, and IL22 (Figure 2) [117]. In imiquimod-treated mice, tapinarof demonstrated less inflammation, epidermal thickening, and reduced proinflammatory cytokines such as IL17, IL19, IL22, IL23, and IL1β [117]. In addition, tapinarof has an antioxidant activity based on inhibition of reactive oxygen species through the stilbene structure and NRF2 pathway [117].

In the phase 2 clinical trial, the PASI75 response to topical tapinarof at week 12 was 65% (1% twice daily) and 56% (1% once daily) when compared to 16% (twice daily) and 5% (once daily) for the placebo. The most frequently reported adverse events were folliculitis and contact dermatitis in the tapinarof groups [118]. Therefore, topical tapinarof is efficacious in the treatment of psoriasis.

## 4. Conclusions

In this review, we summarized new insights regarding the pathogenesis of psoriasis, as it relates to AMPs, DCs, the IL23/IL17 axis, and AhR. Moreover, we summarized new treatments, including JAK inhibitors, ROCK inhibitors, S1P agonists, and AhR agonists. Some of these treatments are currently undergoing clinical trials and are expected to be on the market. To improve the quality of life of psoriatic patients, the choice of available treatments is now increasing.

## Figures and Tables

**Table 1:** Summary of pathogenesis of psoriasis.

Cell Type	Description
KCs	KCs produce AMPs such as β-defensins, S100 proteins, and cathelicidin
pDCs	pDCs stimulated by LL37-DNA complexes produce IFNα
mDCs	mDCs stimulated by LL37-RNA complexes produce TNFα, IL23, and IL12.
LCs	LCs produce IL23
Th17 cells	Th17 cells stimulated by IL23 produce IL17 and IL22 through JAK-STAT pathway IL17 recruits neutrophil and proliferates KCs
VECs	AhR in cutaneous VECs downregulate neutrophil recruitment

KC: keratinocyte; AMP: antimicrobial peptides; pDC: plasmacytoid dendritic cell; IFN: interferon; mDC: myeloid dendritic cell; iDC: inflammatory dendritic cell; TNF: tumor necrosis factor; IL: interleukin; JAK: Janus kinase; STAT: signal transducer and activator of transcription; LC, Langerhans cell; AhR: aryl hydrocarbon receptor; VEC: vascular endothelial cell; Neu: neutrophil; HLA: human leukocyte antigen.

**Table 2:** Summary of new treatments under clinical trials for psoriasis.

Name	Target	Stage	Dosage Form
VTP-43742	RORγt inhibitor	Phase 2	Oral
GSK2981278	RORγ inverse agonist	Phase 1	Topical
BI 655130	IL36-receptor antagonist	Phase 1	Intravenous
Tofacitinib	JAK inhibitor	Phase 3	Oral
Baricitinib	JAK1/JAK2 inhibitor	Phase 2	Oral
BMS-986165	TYK2 inhibitor	Phase 2	Oral
PF-06700841	TYK2/JAK1 inhibitor	Phase 2	Topical
Ponesimod	S1PR1 agonist	Phase 2	Oral
KD025	ROCK2 inhibitor	Phase 2	Oral
Tapinarof	AhR agonist	Phase 2	Topical
